# Investigating the Effect of Hospital Infection Control Informatization on Optimizing Microbiological Specimen Submission Before Antibiotic Therapy: Failure Mode and Effects Analysis

**DOI:** 10.2196/78118

**Published:** 2026-03-10

**Authors:** Jianxiong Wu, Weijiang Zhan, Jun Hua, Qinling Ge, Yuexian Zhu, Huixian Yu, Min Zhao, Xiaoyan Zhan, Bingwei Zhu, Tongqi Xiang, Longxi Lu, Tieying Dai

**Affiliations:** 1 Department of Public Health, The First Affiliated Hospital of Zhejiang Chinese Medical University (Zhejiang Provincial Hospital of Chinese Medicine), 54 Youdian Road, Xihu District, Hangzhou, Zhejiang, 310003, China, 86 18767135989; 2 Department of Computer Center, the First Affiliated Hospital of Zhejiang Chinese Medical University (Zhejiang Provincial Hospital of Chinese Medicine), Hangzhou, Zhejiang, China; 3 Department of Nursing, the First Affiliated Hospital of Zhejiang Chinese Medical University (Zhejiang Provincial Hospital of Chinese Medicine), Hangzhou, Zhejiang, China; 4 Department of Pharmacy, the First Affiliated Hospital of Zhejiang Chinese Medical University (Zhejiang Provincial Hospital of Chinese Medicine), Hangzhou, Zhejiang, China; 5 Discipline Inspection Commission Office, the First Affiliated Hospital of Zhejiang Chinese Medical University (Zhejiang Provincial Hospital of Chinese Medicine), Hangzhou, Zhejiang, China; 6 Microbiology Laboratory, the First Affiliated Hospital of Zhejiang Chinese Medical University (Zhejiang Provincial Hospital of Chinese Medicine), Hangzhou, Zhejiang, China; 7 Logistics Support Department, the First Affiliated Hospital of Zhejiang Chinese Medical University (Zhejiang Provincial Hospital of Chinese Medicine), Hangzhou, Zhejiang, China; 8 Department of Environmental Health, Zhejiang Provincial Center for Disease Control and Prevention, Hangzhou, Zhejiang, China

**Keywords:** microbiological specimen submission, failure mode and effects analysis, antibiotic stewardship, hospital informatization, risk management

## Abstract

**Background:**

Antimicrobial resistance (AMR) poses a critical global health threat, with inappropriate antibiotic use being a major driver. Timely microbiological specimen submission before initiating antibiotic therapy is a cornerstone of antimicrobial stewardship (AMS), enabling pathogen-directed therapy and reducing unnecessary broad-spectrum exposure. However, suboptimal compliance remains common due to workflow interruptions, technological barriers, and behavioral factors. Failure Mode and Effects Analysis (FMEA), a proactive risk-assessment method widely used in health care quality improvement, provides a systematic framework to identify process vulnerabilities and prioritize corrective actions. Despite its increasing application, few studies have integrated FMEA with hospital informatization to optimize microbiological specimen submission workflows in routine AMS practice.

**Objective:**

This study aimed to systematically identify workflow risks affecting preantibiotic microbiological specimen submission and to design, implement, and evaluate informatization-enabled interventions using an FMEA-based framework.

**Methods:**

FMEA was conducted at a tertiary hospital in China. A multidisciplinary team identified potential failure modes across 4 domains: health information systems, personnel, administration, and external support. Risk Priority Numbers (RPNs) and Action Priority (AP) indices were calculated for each failure mode. Targeted interventions were implemented, including dual-verification barcode scanning, artificial intelligence-driven clinical decision support alerts, EHR-integrated training modules, and automated compliance dashboards. Pre- and postintervention specimen submission rates (January 2024-December 2024) were analyzed using the Mann-Kendall trend test.

**Results:**

The top 5 failure modes included PDA barcode scanning failures (RPN=175), inadequate clinical decision support (RPN=140), insufficient clinician awareness (RPN=56), suboptimal oversight mechanisms, and patient-related barriers. Postintervention, significant upward trends were observed in overall specimen submission rates (*P*<.001), with similar improvements for restricted-use (*P*<.001) and special-use antibiotics (*P*<.001).

**Conclusions:**

FMEA-based risk management combined with hospital informatization effectively optimized specimen submission workflows. Real-time decision support, process standardization, and interdisciplinary collaboration significantly enhanced compliance. Future research should evaluate long-term impacts on AMR reduction and diagnostic integration. Integrating FMEA with hospital informatization effectively strengthened the microbiological specimen submission process. Digital decision support, standardized workflows, and real-time monitoring substantially improved compliance with preantibiotic specimen submission. This approach provides an actionable model for data-driven AMS enhancement. Future studies should assess scalability, cost-effectiveness, and impacts on downstream AMR outcomes.

## Introduction

Antimicrobial resistance (AMR) poses a critical threat to global public health and health care systems [1,2]. It was estimated that AMR was associated with 4.71 million deaths worldwide in 2021 [3]. Inappropriate use of antibiotics is a primary driver of AMR [4]. Accurate microbiological pathogen detection is essential for guiding rational antibiotic therapy [5]. On one hand, identifying the causative pathogens and their antimicrobial susceptibility profiles can reduce unnecessary exposure to broad-spectrum antibiotics. On the other hand, microbiological test results support clinicians in refining therapeutic strategies, thereby shortening the infection control cycle. However, multiple studies have reported low rates of microbiological specimen submission prior to initiating antibiotic therapy. A multicenter study in Europe found that only 50% of patients had specimens submitted before antibiotic administration [6]. Similarly, a study conducted in Beijing, China, showed that among patients receiving carbapenem therapy, the preantibiotic microbiological submission rate was only 64.76% [7].

The accuracy and timeliness of pathogen detection rely heavily on standardized and efficient specimen submission workflows. Many countries have established tailored guidelines and policies [8]. Specimens should be obtained prior to initiating antibiotic therapy to ensure timely microbiological culture [9]. For example, China has included the microbiological specimen submission rate for high-priority antibiotics as a core quality indicator in *Healthcare-Associated Infection Management Quality Control Indicators (2024 Edition*) [10]. In clinical practice, multiple challenges hinder preantibiotic specimen submission, including insufficient awareness among clinicians, delays in specimen collection by nursing staff, and transport inefficiencies [11]. As the specimen collection and submission process involves multiple departments and procedural steps, its complexity has hindered improvement of submission rates [12]. Currently, there is a limited understanding of risk factors across the entire microbiological specimen submission pathway.

Failure mode and effects analysis (FMEA), as a risk management tool, has been extensively used in research on optimizing and refining hospital management processes [13,14]. The core principle of FMEA involves assembling an interdisciplinary team to systematically identify potential failure modes within a workflow, analyze their causes and consequences, and prioritize high-risk steps by calculating the Risk Priority Number (RPN), thereby guiding the development of targeted improvement interventions. For example, Yang et al [15] applied FMEA to hospital infection prevention in a stomatology department by mapping the clinical workflow, identifying potential failure modes through expert consensus, and ranking high-risk steps using RPN to inform targeted control strategies. Chiereghin et al [16] used FMEA to improve the quality of an organized colorectal cancer screening program. These studies highlight FMEA’s value in systematically dissecting complex clinical processes and guiding resource allocation toward high-impact improvements. However, its application to microbiological specimen submission within antimicrobial stewardship programs remains limited, particularly when integrated with hospital digital infrastructure.

Digital health and information systems can support this process by embedding real-time alerts into electronic prescribing workflows, prompting clinicians to submit microbiological specimens before initiating antibiotic therapy. Integrated informatics tools, such as electronic health records (EHRs), computerized physician order entry, barcode-based specimen tracking systems, and clinical decision support systems (CDSS), enable end-to-end, closed-loop management of specimen collection, transport, and laboratory processing. However, digital health and information systems may also impose additional workload on health care professionals [17]. Therefore, developing feasible implementation strategies and continuously evaluating the real-world performance of these technologies are essential to ensure sustainable optimization.

Therefore, this study aimed to identify the failure modes contributing to low microbiological specimen submission rates in a tertiary hospital of Chinese Medicine using FMEA and to develop and evaluate targeted interventions to improve specimen submission practices.

## Methods

### Study Setting and Design

**Figure 1.**
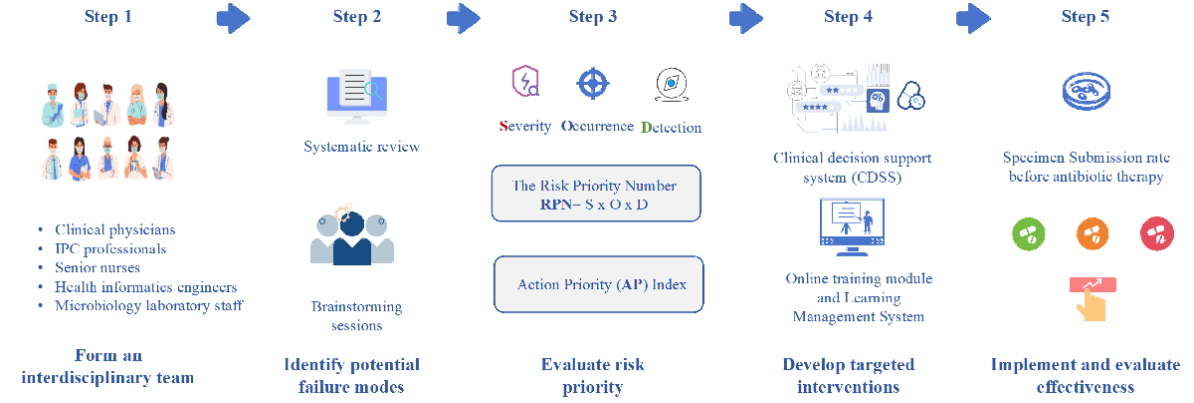
Schematic figure of Failure Mode and Effects Analysis for microbiological specimen submission. IPC: infection prevention and control.

This study was conducted at Zhejiang Provincial Hospital of Chinese Medicine, a general tertiary hospital located in Hangzhou, Zhejiang Province, China. Applying FMEA to assess risks in preantibiotic therapy microbiological specimen submission, formulate targeted interventions, and evaluate their effectiveness. A complete checklist is available in Checklist 1. The implementation steps are as follows (Figure 1):

### Step 1: Form an Interdisciplinary Team

A multidisciplinary expert team was established to ensure comprehensive identification of workflow risks across clinical, laboratory, managerial, and informatics domains. The team consisted of 9 members, including clinical physicians, Disease Prevention and Control Experts, infection prevention and control (IPC) professionals, microbiology laboratory staff, senior nurses, and health informatics engineers. Team members were selected based on predefined criteria: (1) at least 5 years of professional experience in their respective fields; (2) direct involvement in antimicrobial stewardship, specimen management, or infection surveillance activities; (3) familiarity with routine workflows and known bottlenecks within the hospital; and (4) willingness to participate in structured meetings, independent FMEA scoring, and consensus discussions. This interdisciplinary composition ensured that all key steps of the specimen submission process, from clinical decision-making to specimen collection, laboratory processing, and information system operation, were adequately represented, providing a balanced and comprehensive perspective for risk analysis.

### Step 2: Identify Potential Failure Modes

We first conducted a systematic review of the literature to identify common influencing factors in the microbiological specimen submission workflow (Multimedia Appendix 1). Commonly reported factors affecting microbiological specimen submission were extracted and categorized into four predefined domains: (1) health information systems factors, (2) personnel-related factors, (3) administrative and management factors, and (4) external support factors. This framework was used to guide subsequent discussions. Building on this framework, the multidisciplinary expert panel participated in 2 rounds of structured brainstorming sessions. During each session, experts were instructed to identify specific, concrete scenarios within each domain that could plausibly lead to specimen submission failure or delay. To enhance consistency, failure modes were defined as observable deviations from the expected workflow (eg, missed test ordering, delayed collection, incomplete data capture), rather than abstract causes or outcomes. All proposed failure modes were collated, merged, and refined by the research team. Redundant items were consolidated, and ambiguous descriptions were clarified through iterative discussion with panel members. Items were retained only if consensus was reached that the scenario was (1) operationally distinct, (2) locally relevant to routine practice in the study hospital, and (3) theoretically supported by either literature evidence or expert experience. A final set of 30 locally relevant failure modes was generated. These items formed the basis for subsequent FMEA scoring and prioritization.

### Step 3: Evaluate Risk Priority

Before formal scoring, all team members received standardized training on the FMEA scoring criteria provided by the research team. Each member independently evaluated the Severity (S), Occurrence (O), and Detection (D) of each potential failure mode. After all 9 members completed their independent assessments, a consensus meeting was convened to discuss discrepancies and clarify scoring decisions. Final S, O, and D scores for each failure mode were determined through group consensus. The RPN was calculated using the formula: RPN=S×O×D.

The scoring range for S, O, and D was 1‐10. Conventionally, corrective actions are mandated when the RPN exceeds 125. To address limitations of the RPN method, we introduced the Action Priority (AP) index. The determination of AP followed the standard methodology outlined in the Automotive Industry Action Group–Verband der Automobilindustrie FMEA guidelines. Unlike the RPN, the AP level is not calculated through a mathematical formula; instead, it is assigned by consulting the guideline’s AP lookup table, which specifies the corresponding action level based on different combinations of Severity (S), Occurrence (O), and Detection (D). As shown in Multimedia Appendix 2, AP levels are categorized as high, medium, or low. High AP indicates that corrective actions should be taken with priority, medium AP suggests that action is recommended but can be scheduled, and low AP indicates that existing controls are acceptable and require only routine monitoring. High-priority failure modes identified by the FMEA (based on both RPN and AP indices).

### Step 4: Develop Targeted Interventions

For each mode, the multidisciplinary team collaboratively designed corresponding digital health solutions. All digital interventions underwent iterative refinement through pilot testing in selected departments. Feedback from clinicians, nurses, infection control practitioners, and IT personnel was collected to optimize alert thresholds, interface design, workflow integration points, and data visualization formats.

The final intervention package consisted of three interrelated components:

1. An artificial intelligence-driven (DeepSeek-R1) CDSS was developed and integrated into the hospital’s EHR and computerized physician order entry system to promote microbiological specimen submission before antibiotic therapy. When a clinician initiates an order for therapeutic antibiotics, particularly restricted-use or special-use agents, the system automatically evaluates whether a corresponding microbiological specimen has been ordered or collected within a predefined time window.
2. Online training module and learning management system (LMS): The module was jointly created by the Infection Prevention and Control Department, the Microbiology Laboratory, and the IT Department. The training curriculum covered standardized procedures for microbiological specimen collection, requirements for specimen transport, electronic order entry workflows, and regulations related to preantibiotic specimen submission.
3. Supplementary digital process-enhancement measures: Additional supportive measures included enhanced barcode-based specimen tracking and monitoring dashboards with feedback mechanisms. Barcode-based tracking strengthened specimen traceability during collection and transport, reducing data loss caused by scanning failures or workflow deviations. Dashboards aggregated compliance data by department and antibiotic category, generating periodic feedback reports for clinical units and infection control teams to facilitate continuous quality improvement.

### Step 5: Implement and Evaluate Effectiveness

After developing the intervention measures, we proceeded with the implementation phase following the standard quality improvement process. The preparatory stages of FMEA, including workflow mapping, identification and scoring of failure modes, prioritization, and intervention design, were completed between January 1 and March 31, 2024. Beginning on April 1, 2024, the FMEA-informed interventions were formally implemented. Physicians, nurses, and support staff received training on the updated specimen submission workflow and information system functions through the online learning platform. The effectiveness evaluation covered the period from January to December 2024. Improvements were assessed by comparing pre- and postintervention microbiological specimen submission rates.

Three core outcome indices were evaluated:

1. Specimen submission rate before antibiotic therapy of inpatients (%)=number of inpatients with pathogen submission before antibiotic therapy/number of inpatients with therapeutic application of antibiotics during the same period *100%.
2. Specimen submission rate before restricted-use antibiotic therapy of inpatients (%)=number of inpatients with pathogen submission before restricted-use antibiotic therapy/number of inpatients with therapeutic application of restricted-use antibiotics during the same period *100%.
3. Specimen submission rate before special-use antibiotic therapy of inpatients (%)=number of inpatients with pathogen submission before special-use antibiotic therapy/number of inpatients with therapeutic application of special-use antibiotics during the same period *100%.

### Data Collection

Specimen submission data from January 1, 2024, to December 31, 2024, was extracted from the Hospital Infection Surveillance System (Xinglin System). The dataset included antibiotic order time, drug category (restricted-use, special-use), and timing of administration relative to specimen submission. To ensure data quality and consistency, only complete records with clearly documented timestamps for both specimen submission and antibiotic prescribing were included in the analysis. Duplicate entries, canceled orders, and specimens rejected by the laboratory due to quality issues were excluded.

### Data Analysis

Statistical analyses were performed using SPSS (version 27.0; IBM Corp). Categorical variables were reported as counts (percentages). The Mann-Kendall trend test was used to examine the trend of submission rate over time. When the *z* score was positive, it meant that the composition ratio of each indicator showed an upward trend. When the *z* score was negative, it meant that the composition ratio of each indicator presented a downward trend. Statistical significance was set at 2-tailed *P*<.05.

### Ethical Considerations

This study was approved by the Ethics Committee of the First Affiliated Hospital of Zhejiang Chinese Medical University (2025-KLS-038-01). As this study used retrospective data, the ethics committee waived the requirement for participants to provide written informed consent. The research data does not involve individual patient information. No compensation was provided to participants as this study was a secondary analysis of preexisting data with no new patient recruitment or intervention. The research was conducted in accordance with the Declaration of Helsinki.

## Results

### Hazard Analysis of Specimen Submission

The top five failure modes limiting preantibiotic specimen submission were (1) PDA barcode scanning failures, (2) inadequate clinical decision support alerts, (3) insufficient clinician awareness of testing protocols, (4) suboptimal oversight mechanisms, and (5) patient-related health constraints (Table 1).

**Table 1.** Failure modes summary for Failure Mode and Effects Analysis.

Potential failure modes	RPN^a^	AP^b^
Health information system		
Interoperability gaps exist between the inpatient electronic medical records system and the health care–associated infection surveillance system, resulting in inaccurate and delayed data sharing and synchronization between the 2 platforms.	146	Moderate
Failure of PDA barcode scanning or over-rapid scanning operations led to unrecorded specimen collection data in the system, compromising preanalytical traceability.	168	High
Incomplete capture or delayed updating of pathogen detection-related data compromised the integrity of microbiological specimen submission records.	158	High
The absence of proactive alerts for pathogen detection failed to prompt clinicians to initiate microbiological testing before antimicrobial therapy, resulting in prescription restrictions due to noncompliance with stewardship protocols.	70	Low
The hospital information system failed to accurately distinguish the purpose of antimicrobial use (therapeutic vs prophylactic) during medication documentation, undermining the precision of stewardship analytics.	35	Low
The calculation methodology for pathogen detection-related metrics in the information system was not aligned with national standards, resulting in inaccurate statistical outcomes.	84	Low
Hospital personnel		
Delayed communication and coordination among IPC^c^ professionals due to ineffective communication channels or the absence of established mechanisms hindered timely interventions.	48	Low
Incomplete data verification by IPC professionals was attributed to the overwhelming volume of data and the inherent limitations of manual review processes, leading to frequent oversights.	32	Low
Misinterpretation of infection protection and control standards by IPC professionals occurred due to frequent updates and inadequate, timely training, resulting in noncompliant practices.	48	Low
Health care providers demonstrated insufficient awareness of pathogen detection protocols, with inadequate understanding of its clinical significance, leading to missed testing opportunities in cases where microbiological analysis was indicated.	126	High
Delays in specimen submission by health care providers were attributed to heavy workloads leading to oversight or missed submissions, as well as suboptimal time window settings that conflicted with clinical workflows.	75	Moderate
Noncompliant specimen collection practices by health care providers, stemming from insufficient familiarity with collection protocols and deviations from aseptic techniques, resulted in specimen contamination or invalid samples.	50	Low
Noncompliant specimen transport practices by health care providers, due to insufficient knowledge of transport protocols and inadequate protective measures during transit, compromised sample integrity.	80	Low
Health care providers mistakenly prescribed therapeutic antibiotics instead of prophylactic regimens, leading to inappropriate antimicrobial use.	80	Low
Ineffective communication between nurses and physicians, due to the absence of established collaboration mechanisms, resulted in suboptimal specimen collection practices, as nurses lacked clear guidance on proper techniques and timing, leading to noncompliant samples.	96	Low
IT personnel failed to facilitate tripartite coordination, lacking effective communication and collaboration mechanisms.	48	Low
IT personnel lacked sufficient knowledge of infection surveillance data extraction guidelines: the complexity of guidelines for building infection surveillance information systems made them difficult to understand.	48	Low
Insufficient expertise among hospital microbiology laboratory personnel and inadequate detection technology levels.	40	Low
Health care administration		
Inadequate regulatory mechanisms for pathogen detection specimen submission: issues include unclear regulatory responsibilities, limited regulatory methods, and insufficient regulatory enforcement.	120	Low
Inadequate performance evaluation mechanisms for pathogen detection specimen submission: evaluation metrics are not linked to health care providers’ performance or career advancement, lacking incentives.	48	Low
Inadequate feedback mechanisms for pathogen detection specimen submission: test results are not timely or accurately communicated to health care providers.	48	Low
Inadequate communication and collaboration mechanisms among hospital departments: information flow and interdepartmental coordination are suboptimal.	96	Low
Inadequate training content on pathogen detection specimen submission: issues include outdated content, lack of relevance, or limited training methods.	84	Low
Lack of standardized specimen collection and submission processes: issues include unclear procedures, inconsistent operational standards, or poor process execution.	96	Low
External support		
Some patients may not understand or be willing to cooperate with pathogen detection specimen submission, leading to difficulties in sample collection.	49	Low
Patients’ poor health status or physical condition may hinder cooperation, affecting the smooth progress of sample collection (eg, difficulty in expectorating sputum leading to challenges in sputum sample collection).	49	Low
Patients’ health status or physical condition may affect the smooth progress of sample collection.	192	Moderate
Limited space in the microbiology laboratory may reduce the efficiency of sample processing. During peak hours, sample backlogs may occur, prolonging testing times.	80	Low
Convenience of specimen collection: health care providers may face space and equipment limitations when collecting samples in patient rooms. For example, the lack of appropriate sputum induction devices may compromise sample quality, reducing motivation for submission.	64	Low
Inadequate cleaning and disinfection of patient rooms: a high pathogen load in the room environment may lead to cross-contamination, interfering with the diagnosis of the patient's own infectious pathogens.	128	Moderate

a RPN: Risk Priority Number.

b AP: action priority.

c IPC: infection prevention and control.

### Develop Targeted Interventions

For the potentially high-risk failure modes, recommended interventions were identified with a view to reducing their occurrence or improving their detection (Table 2). An LMS-based online training was implemented. All personnel involved in specimen collection or antibiotic order entry were required to complete the module and pass a competency assessment. A score of at least 80% was considered a passing result. The LMS automatically recorded training completion and assessment outcomes; individuals who did not complete the module or did not achieve a passing score were required to undergo retraining before being allowed to independently perform the related tasks. A real-time CDSS was embedded into the antibiotic prescribing process.

**Table 2.** Interventions recommended to improve the specimen submission rate.

High-risk failure modes	Targeted interventions
PDA barcode scanning failures	Implement dual-verification protocols combining manual entry with barcode scanning, accompanied by real-time error alerts in the mobile application.
Inadequate clinical decision support alerts	Deploy AI^a^-driven decision support systems to automatically flag high-risk cases requiring preantimicrobial testing, with escalating alert tiers.
Insufficient clinician awareness of testing protocols	Develop role-specific e-Learning modules with embedded competency assessments. Integrate protocol reminders into electronic health record order entry workflows.
Suboptimal oversight mechanisms	Establish automated dashboards tracking testing compliance, with weekly feedback reports to department heads. Introduce “process champions” in each unit to monitor adherence and facilitate peer-to-peer learning.
Patient-related health constraints	Create adaptive sampling protocols. Train nursing staff in motivational interviewing techniques to improve patient engagement.

a AI: artificial intelligence.

When clinicians initiated orders for therapeutic antibiotics, particularly restricted-use or special-use agents, the CDSS automatically evaluated whether an appropriate microbiological specimen had been ordered. If noncompliance was detected, the system generated real-time alerts prompting clinicians to initiate specimen submission. This workflow enabled prospective intervention at the point of care, reducing missed submissions caused by time pressure or oversight. Supportive process-enhancement measures were integrated to reinforce execution and oversight. Enhanced PDA-based barcode scanning strengthened specimen traceability during collection and transport, reducing data loss related to scanning failures or rapid operations. In parallel, automated monitoring dashboards aggregated specimen submission compliance data by department and antibiotic category. Regular feedback reports were provided to clinical units and infection control teams, supporting targeted supervision and continuous workflow optimization.

### Effect of FMEA Management on Specimen Submission

**Figure 2.**
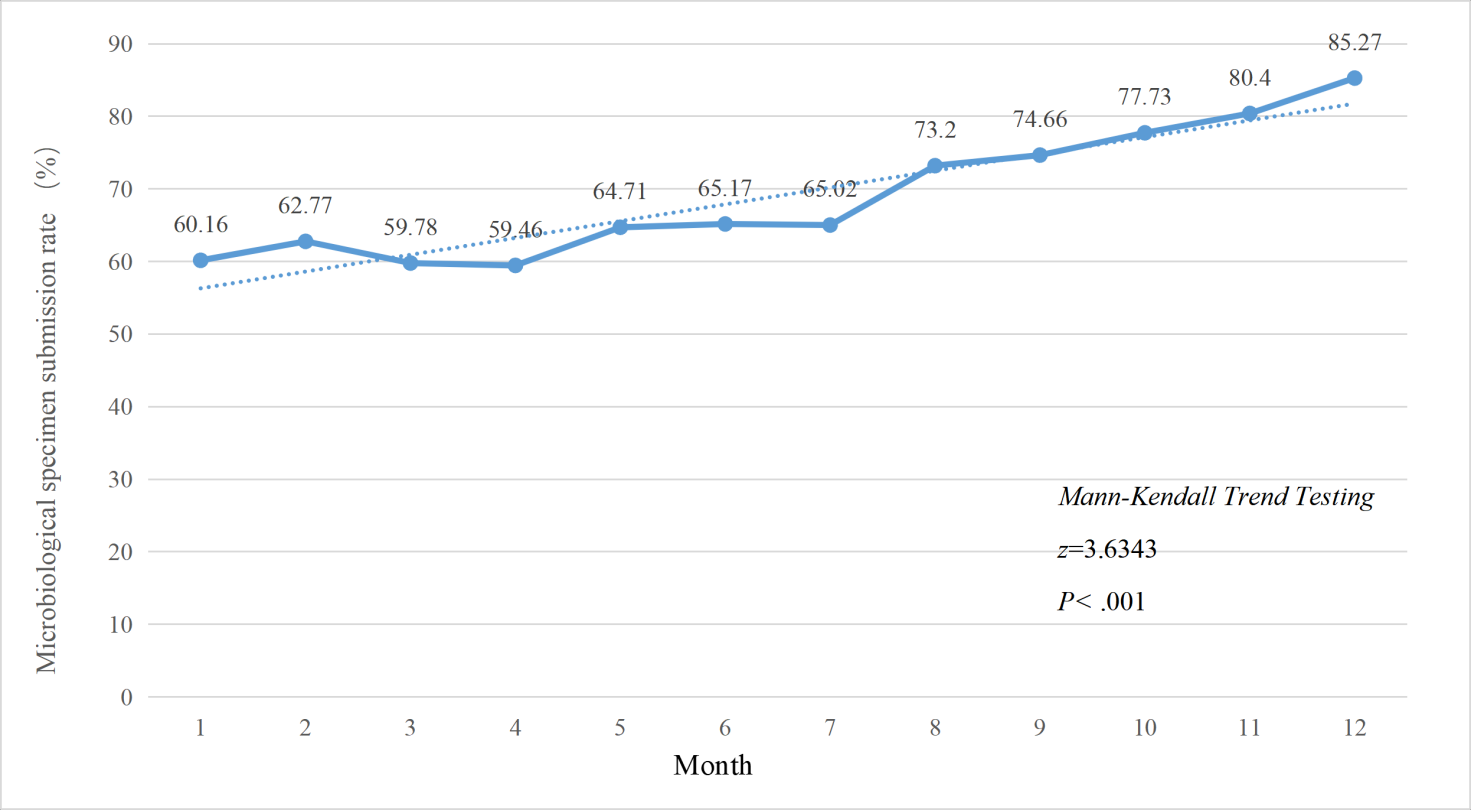
Changes in the specimen submission rate before antibiotic therapy among in-patients, 2024.

**Figure 3.**
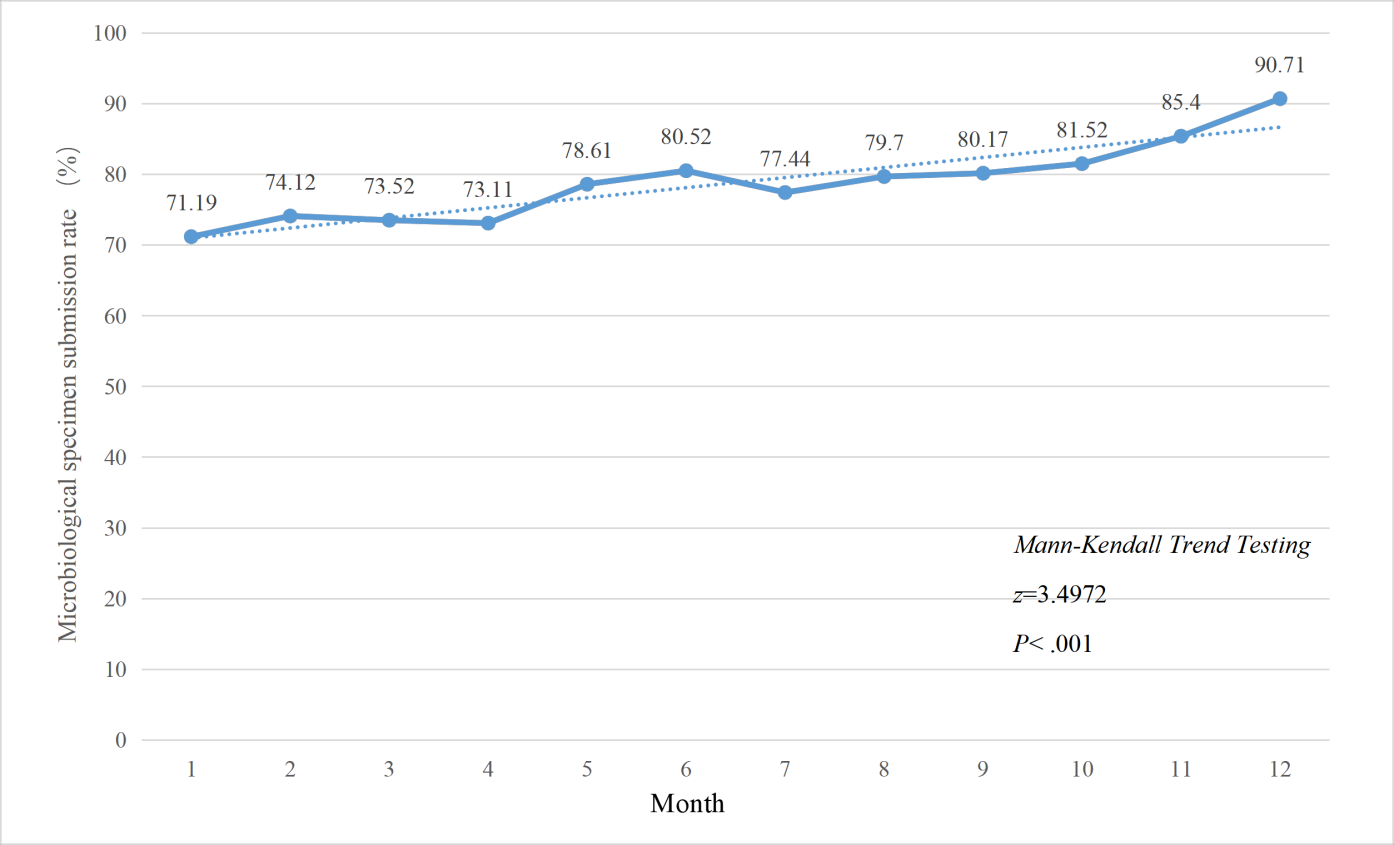
Changes in the specimen submission rate before restricted-use antibiotic therapy among in-patients, 2024.

**Figure 4.**
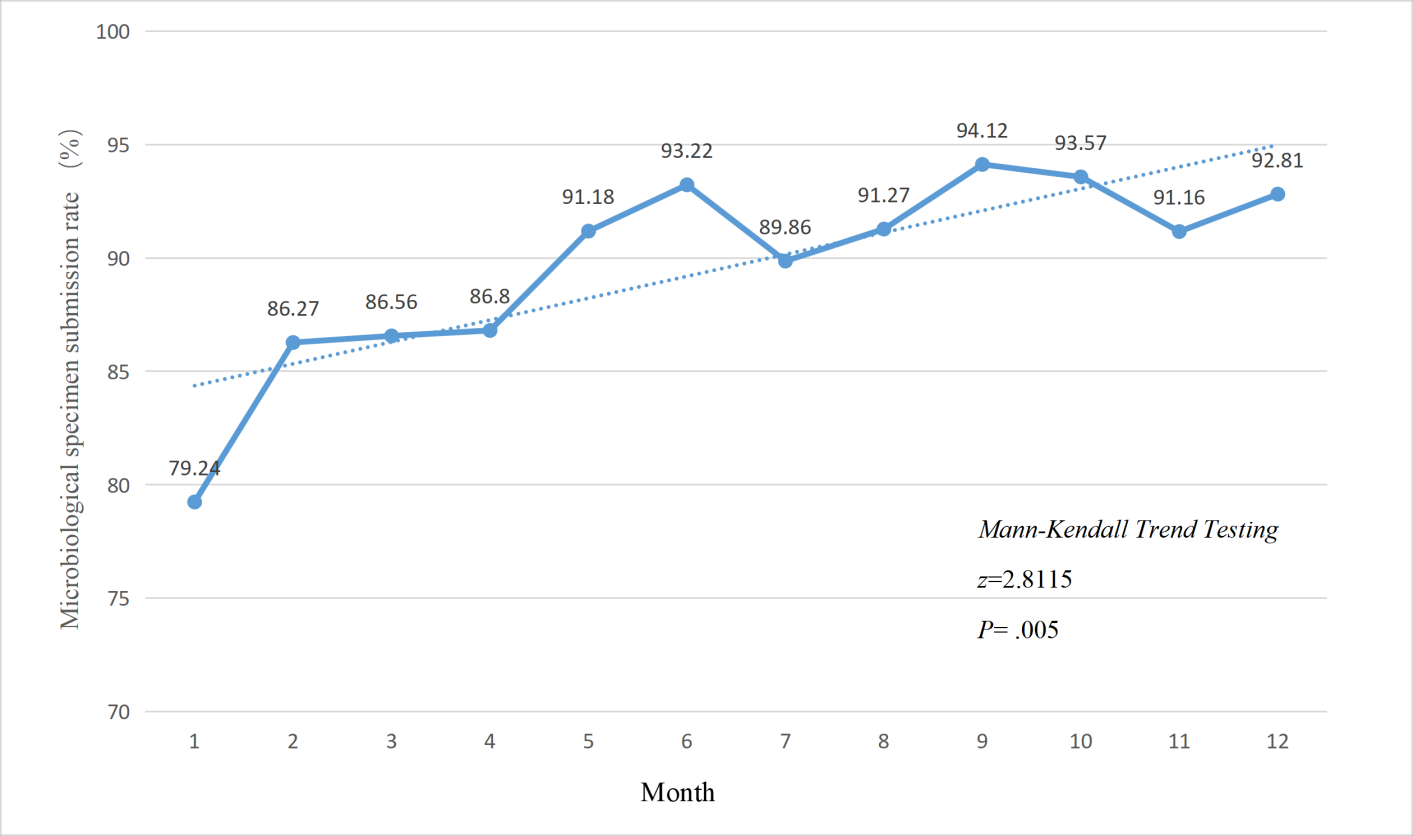
Changes in the specimen submission rate before special-use antibiotic therapy among in-patients, 2024.

Following the implementation of FMEA management in April 2024, significant improvements were observed in the overall specimen submission rate (Figure 2), the submission rate for restricted antibiotics (Figure 3), and the submission rate for special-use antibiotics (Figure 4). The Mann-Kendall trend test revealed that all 3 categories of specimen submission rates exhibited a statistically significant upward trend (*P*<.001).

## Discussion

### Principal Findings

Microbiological specimen submission plays a pivotal role in hospital infection control and promoting rational antibiotic use, representing a complex systemic endeavor [18,19]. This study implemented the internationally recognized risk assessment tool—FMEA, to conduct a multidimensional evaluation of the entire etiological specimen submission workflow, spanning information systems, health care personnel, hospital administration, and external support. Through expert panel discussions, the research identified multiple failure modes and prioritized targeted intervention strategies, providing scientifically grounded decision-making support for improving the quality of specimen submission practices. This study provides a replicable pathway and methodological framework for improving microbiological specimen submission workflows, and may indirectly support hospital infection prevention efforts by enhancing antimicrobial stewardship and strengthening infection surveillance.

The advancement of intelligent health care information systems has emerged as a critical driver for improving specimen submission rates [20]. FMEA-based investigation identified PDA barcode scanning failures and insufficient CDSS alerts as high-priority intervention targets. Mechanistically, real-time alert mechanisms within information systems can trigger specimen collection reminders before antimicrobial administration, thereby minimizing workflow delays caused by human oversight and ensuring precise temporal control of submissions [21]. Concurrently, integrated multisource data platforms, by consolidating heterogeneous data from EHRs, laboratory results, and other sources, enable dynamic decision-making models that assist clinicians in accurately evaluating the necessity of testing indications and the specificity of required assays [22]. These findings reveal a dual-driven mechanism of information system optimization: process monitoring reduces operational deviations, while data integration enhances diagnostic decision-making efficacy.

This study also revealed that inadequate clinician awareness of specimen submission constitutes a critical bottleneck in improving submission quality. To address this, the research team implemented a dual-track capacity-building framework, “theoretical training+practical supervision,” alongside intelligent system upgrades: standardized operational training was used to reinforce protocol adherence, while interdepartmental supervision mechanisms established closed-loop quality improvement cycles. Postintervention evaluation demonstrated statistically significant improvements in specimen submission rates (*P*<.001). Although the observational study design precludes causal inferences regarding direct relationships between interventions and outcome improvements, the synergistic decline across multidimensional surveillance metrics indirectly suggests potential systemic spillover effects of FMEA-based management in hospital infection prevention and antimicrobial stewardship.

Despite achieving improvements in nosocomial infection specimen submission processes and promoting rational antimicrobial use through hospital-wide informatization, several areas for development remain. Externally, budget constraints limit access to advanced yet costly testing modalities, hindering comprehensive testing. Variations in patient case mix create department-specific testing demands that cannot be uniformly addressed. Additionally, diagnosis-related group reimbursement constraints introduce cost-benefit trade-offs that indirectly influence testing decisions. Therefore, hospital management must continue refining collaborative mechanisms across clinical, technical, and administrative departments. Strategic initiatives should include optimizing test menu configurations through cost-effectiveness analysis, developing disease-specific testing guidelines, and leveraging informatization for continuous monitoring to enhance staff efficiency. These measures will ultimately strengthen AMR control, promote rational antibiotic use, improve surveillance capabilities for emerging pathogens, and drive health care quality toward higher precision and patient safety.

This study has several limitations that warrant consideration. First, some of the interventions relied on a relatively well-developed hospital information infrastructure, such as artificial intelligence-driven CDSS alerts. Institutions with limited digital capacity may need to adopt manual checklists or simplified reminder mechanisms as alternatives. Second, the study was conducted in a tertiary TCM hospital. Although the hospital includes Western medicine departments such as the intensive care unit, respiratory medicine, and emergency medicine, with antimicrobial use patterns comparable to general hospitals, the patient population and service model of TCM hospitals may differ from other health care settings, which may limit generalizability. Nevertheless, the methodological framework of FMEA is highly transferable, and individual institutions may adapt specific interventions according to their local resources and workflows. Additionally, this was a single-center, before and after study without a control group, which imposes inherent limitations on causal inference. Improvements in specimen submission rates may not be attributable solely to the FMEA-based interventions; they may also reflect the hospital’s broader emphasis on antimicrobial stewardship, underlying secular trends in workflow optimization, or concurrent quality improvement initiatives or policy changes. Future research should validate the effectiveness of these interventions using multicenter designs with appropriate control groups.

### Conclusions

This study validated FMEA as a risk management tool for optimizing preantimicrobial specimen submission workflows. By addressing critical failures like barcode errors and inadequate decision support, FMEA-based interventions significantly improved specimen submission rates for restricted/special-use antibiotics, aligning with national quality standards. Challenges, including cost constraints and patient barriers, necessitate policy-level solutions. This model offers a scalable framework for antimicrobial stewardship, with future research needed to evaluate long-term outcomes and advanced diagnostic integration.

## Supplementary material

10.2196/78118 Multimedia Appendix 1 Systematic review of the submission rate of microbiological specimen before antibiotic therapy.

10.2196/78118 Multimedia Appendix 2 Action Priority lookup table.

10.2196/78118 Checklist 1 iCHECK-DH (Guidelines and Checklist for the Reporting on Digital Health Implementations) guidelines and checklist for the reporting on digital health implementations.

## Abbreviations

AMR: antimicrobial resistance
AP: Action Priority
CDSS: clinical decision support systems
EHR: electronic health record
FMEA: Failure Mode and Effects Analysis
LMS: learning management system
RPN: Risk Priority Number
TCM: traditional Chinese medicine
