# Supplementary material for: Investigating the Effect of Hospital Infection Control Informatization on Optimizing Microbiological Specimen Submission Before Antibiotic Therapy: Failure Mode and Effects Analysis
Source: J Med Internet Res. 2026 Mar 10;28:e78118. doi: 10.2196/78118 (PMC12974997; doi:10.2196/78118)

**Search Strategy**

A thorough review of the literature was performed using PubMed, and China National Knowledge Infrastructure (CNKI).

**Table S1.** Search strategy.

| **Database** | **Search Query** | **Results** |
| --- | --- | --- |
| PubMed | #1 "antibiotic therapy" OR "antimicrobial stewardship" OR "antimicrobial utilization" OR "appropriate use" | 48,120 |
|  | #2 "Microbiological Techniques"[Mesh] OR "Specimen Handling"[Mesh] OR "microbiological specimen*" OR "culture submission" OR "blood culture*" OR "specimen collection" OR "diagnostic specimen*" OR "pathogenic test" OR "pathogen detection" | 520,906 |
|  | #3 (#1) AND (#2)  Filters applied: Humans, English, from 2000/01/01 - 2025/03/01 | 5,608 |
| CNKI | #1 TKA='抗生素治疗' + '抗菌药物管理' + '抗菌药物治疗' + '抗菌素治疗' （同义词扩展） | 32,118 |
|  | #2 TKA='病原学送检' + '病原学检测' + '微生物送检' + '微生物检测' （同义词扩展） | 39,662 |
|  | #4 #1 and #2（同义词扩展）  发表时间：2000-01-01 – 2025-03-01 | 520 |

**Figure S1**. Flowchart of literature screen.


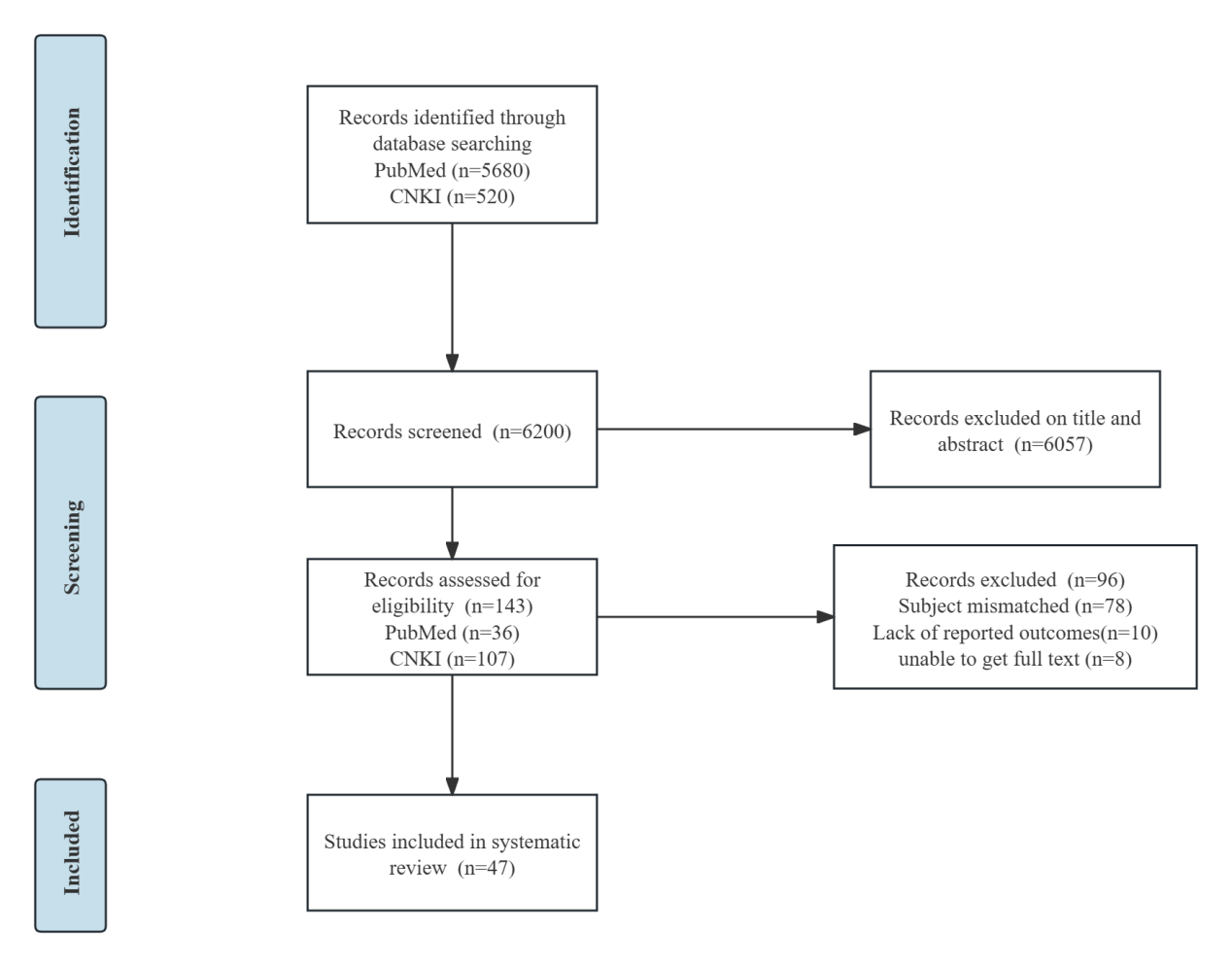

Supplement: Multimedia Appendix 1 [file jmir-v28-e78118-s001.docx]
