# Supplementary material for: Investigating the Effect of Hospital Infection Control Informatization on Optimizing Microbiological Specimen Submission Before Antibiotic Therapy: Failure Mode and Effects Analysis
Source: J Med Internet Res. 2026 Mar 10;28:e78118. doi: 10.2196/78118 (PMC12974997; doi:10.2196/78118)
Supplement: Multimedia Appendix 2 [file jmir-v28-e78118-s002.docx]

**Table S1.** Action Priority (AP) evaluation criteria.

| **Severity** | **S** | **Occurrence** | **O** | **Detection,** | **D** | **Priority for Action (AP)** |
| --- | --- | --- | --- | --- | --- | --- |
| Very high | 9,10 | Very high | 8-10 | Low –  Very low | 7-10 | High |
|  |  |  |  | Medium | 5,6 | High |
|  |  |  |  | High | 2-4 | High |
|  |  |  |  | Very high | 1 | High |
|  |  | High | 6,7 | Low –  Very low | 7-10 | High |
|  |  |  |  | Medium | 5,6 | High |
|  |  |  |  | High | 2-4 | High |
|  |  |  |  | Very high | 1 | High |
|  |  | Medium | 4,5 | Low –  Very low | 7-10 | High |
|  |  |  |  | Medium | 5,6 | High |
|  |  |  |  | High | 2-4 | High |
|  |  |  |  | Very high | 1 | Medium |
|  |  | Low | 2,3 | Low –  Very low | 7-10 | High |
|  |  |  |  | Medium | 5,6 | Medium |
|  |  |  |  | High | 2-4 | Low |
|  |  |  |  | Very high | 1 | Low |
|  |  | Very low | 1 | Very high - Very low | 1-10 | Low |
| High | 7,8 | Very high | 8-10 | Low –  Very low | 7-10 | High |
|  |  |  |  | Medium | 5,6 | High |
|  |  |  |  | High | 2-4 | High |
|  |  |  |  | Very high | 1 | High |
|  |  | High | 6,7 | Low –  Very low | 7-10 | High |
|  |  |  |  | Medium | 5,6 | High |
|  |  |  |  | High | 2-4 | High |
|  |  |  |  | Very high | 1 | Medium |
|  |  | Medium | 4,5 | Low –  Very low | 7-10 | High |
|  |  |  |  | Medium | 5,6 | Medium |
|  |  |  |  | High | 2-4 | Medium |
|  |  |  |  | Very high | 1 | Medium |
|  |  | Low | 2,3 | Low –  Very low | 7-10 | Medium |
|  |  |  |  | Medium | 5,6 | Medium |
|  |  |  |  | High | 2-4 | Low |
|  |  |  |  | Very high | 1 | Low |
|  |  | Very low | 1 | Very high- Very low | 1-10 | Low |
| Medium | 4-6 | Very high | 8-10 | Low- Very low | 7-10 | High |
|  |  |  |  | Medium | 5,6 | High |
|  |  |  |  | High | 2-4 | Medium |
|  |  |  |  | Very high | 1 | Medium |
|  |  | High | 6,7 | Low- Very low | 7-10 | Medium |
|  |  |  |  | Medium | 5,6 | Medium |
|  |  |  |  | High | 2-4 | Medium |
|  |  |  |  | Very high | 1 | Low |
|  |  | Medium | 4,5 | Low- Very low | 7-10 | 中 |
|  |  |  |  | Medium | 5,6 | Low |
|  |  |  |  | High | 2-4 | Low |
|  |  |  |  | Very high | 1 | Low |
|  |  | Low | 2,3 | Low- Very low | 7-10 | Low |
|  |  |  |  | Medium | 5,6 | Low |
|  |  |  |  | High | 2-4 | Low |
|  |  |  |  | Very high | 1 | Low |
|  |  | Very low | 1 | Very high- Very low | 1-10 | Low |
| Low | 2,3 | Very high | 8-10 | Low | 7-10 | Medium |
|  |  |  |  | Low | 5,6 | Medium |
|  |  |  |  | High | 2-4 | Low |
|  |  |  |  | Very high | 1 | Low |
|  |  | High | 6,7 | Low | 7-10 | Low |
|  |  |  |  | Low | 5,6 | Low |
|  |  |  |  | High | 2-4 | Low |
|  |  |  |  | Very high | 1 | Low |
|  |  | Medium | 4,5 | Low –  Very low | 7-10 | Low |
|  |  |  |  | Medium | 5,6 | Low |
|  |  |  |  | High | 2-4 | Low |
|  |  |  |  | Very high | 1 | Low |
|  |  | Low | 2,3 | Low-Very low | 7-10 | Low |
|  |  |  |  | Medium | 5,6 | Low |
|  |  |  |  | High | 2-4 | Low |
|  |  |  |  | Very high | 1 | Low |
|  |  | Very low | 1 | Very high- Very low | 1-10 | Low |
| Very low | 1 | Very low- Very high | 1-10 | Very high- Very low | 1-10 | Low |
